# Elevation of HO-1 Expression Mitigates Intestinal Ischemia-Reperfusion Injury and Restores Tight Junction Function in a Rat Liver Transplantation Model

**DOI:** 10.1155/2015/986075

**Published:** 2015-05-10

**Authors:** Xinjin Chi, Weifeng Yao, Hua Xia, Yi Jin, Xi Li, Jun Cai, Ziqing Hei

**Affiliations:** ^1^Department of Anesthesiology, Third Affiliated Hospital, Sun Yat-Sen University, Guangzhou, Guangdong 510630, China; ^2^Department of Anesthesiology, The Affiliated Hospital of Luzhou Medical College, Luzhou, Sichuan 646000, China; ^3^Department of Pathology, Third Affiliated Hospital, Sun Yat-Sen University, Guangzhou, Guangdong 510630, China; ^4^Department of Breast and Thyroid Surgery, Third Affiliated Hospital, Sun Yat-Sen University, Guangzhou, Guangdong 510630, China

## Abstract

*Aims*. This study was aimed at investigating whether elevation of heme oxygenase-1 (HO-1) expression could lead to restoring intestinal tight junction (TJ) function in a rat liver transplantation model. *Methods*. Intestinal mucosa injury was induced by orthotopic autologous liver transplantation (OALT) on male Sprague-Dawley rats. Hemin (a potent HO-1 activator) and zinc-protoporphyrin (ZnPP, a HO-1 competitive inhibitor), were separately administered in selected groups before OALT. The serum and intestinal mucosa samples were collected at 8 hours after the operation for analysis. *Results*. Hemin pretreatment significantly reduced the inflammation and oxidative stress in the mucosal tissue after OALT by elevating HO-1 protein expression, while ZnPP pretreatment aggravated the OALT mucosa injury. Meanwhile, the restriction on the expression of tight junction proteins zonula occludens-1 and occludin was removed after hemin pretreatment. These molecular events led to significant improvement on intestinal barrier function, which was proved to be through increasing nuclear translocation of nuclear factor-E2-related factor 2 (Nrf2) and reducing nuclear translocation of nuclear factor kappa-B (NF-*κ*B) in intestinal injured mucosa. *Summary*. Our study demonstrated that elevation of HO-1 expression reduced the OALT-induced intestinal mucosa injury and TJ dysfunction. The HO-1 protective function was likely mediated through its effects of anti-inflammation and antioxidative stress.

## 1. Introduction

For more than 30 years liver transplantation has been an effective treatment procedure for end-stage liver diseases [1, 2]. One- and five-year survival rate following liver transplantation are 90% and 75%, respectively [3]. One of challenges during liver transplantation is the organ injury. Liver is particularly susceptible to ischemia-reperfusion injury as a result of storage, transportation, and operational procedure from donor to recipient [4]. Indeed, acute organ injury leads to poor early recovery in recipients. Organ injury is not limited to the liver. We have demonstrated that the intestine injury occurred in a rat orthotopic autologous liver transplantation (OALT) model [5]. Intestine injury can lead to the spread of intraluminal bacteria and endotoxin to organs and tissues beyond the intestine. Diffusion of bacteria and toxins is a primary cause of systemic inflammatory response syndrome (SIRS), MODS, and multiple organ failure [6, 7].

During liver transplantation procedure, the recipients inevitably experienced a short anhepatic phase (including occlusion of portal vein that leads to intestine congestive ischemia) and gastrointestinal congestion for about 45–65 min or longer [8]. The epithelial cells of the intestinal mucosa are sensitive to the hypoxia-ischemia upon intestinal congestion, and they are often subjected to apoptosis and necrosis after the surgery [9]. Meanwhile, the transplanted liver also inevitably underwent ischemia-reperfusion injury during the process. These factors are causatively associated with postoperative complications including the damage to intestinal barrier and the barrier dysfunction, and the increase of the postoperative mortality rate. However, the mechanisms underlying intestinal barrier dysfunction during the perioperative period remain much unknown.

Tight junction (TJ) refers to the closely shared areas between two neighboring cells where membranes at both sides join together to form a virtually impermeable barrier. Compromised TJ barrier structure increases paracellular permeability. The resulting release of proinflammatory molecules within GI track could trigger mucosal immune system activation, leading to tissue damage consisting of ongoing inflammatory reaction and oxidative stress. Evidence from clinical and experimental studies indicated that alteration of intestinal TJ barrier is critical in leading to intestinal dysfunction [10–12].

Heme oxygenase (HO) is an enzyme specialized in degrading heme and is assembled with biliverdin, CO [13], and free iron [14]. HO-1 is normally expressed in the mucosal layer of gastrointestinal track [15, 16]. HO-1 expression is upregulated rapidly to respond to stress and injury in the mucosa [17]. Indeed, many studies have shown that HO-1 exerted protective functions against stress-related tissue damage [17–19]. The upregulation of HO-1 upon stress suggests that HO-1 is a part of intrinsic network to mitigate tissue injury in the gastrointestinal tract [20].

Upon intestine injury detection of metabolic products released by overgrown bacteria is useful for diagnosing the intestinal barrier disorder. D-Lactate (D-LA) is a product released by many of microflora resided in the human gastrointestinal tract. Thus increase of serum D-LA level is a sign of intestinal ischemia [21–23]. Diamine oxidase (DAO) is only synthesized in epithelial cells of intestinal villi which are usually an epicenter of the ischemia. Released DAO by damaged mucosal cells will increase its serum concentration. Intestinal-type fatty acid-binding protein (FABP2) is exclusively released by the damaged intestinal epithelial cells [24]. Intestinal mucosal barrier is a protective complex structure primarily containing tight junctions (TJs); TJs seal individual mucosal membranes at conjunction. The integrity of TJs is the foundation of the structural stability and functional effectiveness of the epithelial barrier [25, 26]. Occludin, tricellulin, claudin, and functional adhesion molecules are internal component of TJs structure [27–29]. It is expected to detect altered levels of those proteins in both serum and injured epithelial cells upon OALT ischemia and other stress [30].

We hypothesized that HO-1 expression was significantly elevated to respond to OALT-induced organ injury. The current study was aimed at investigating whether elevation of HO-1 expression could restore intestinal barriers function through protecting TJ structure and if anti-inflammation and antioxidative stress pathway was the mechanistic contributor to generating protective function in a rat liver transplantation model.

## 2. Methods and Materials

### 2.1. Animals

Male Sprague-Dawley rats (weighing 200–250 g) were cohosted in individual cages in a temperature-controlled room with a specific pathogen-free, laminar flow atmosphere in the Department of Laboratory Animal Center at Sun Yat-Sen University. The animal room was switched to dark after 12 hr light each day. They were given a week to get accustomed to environment before the experiment. Rats were allowed to drink water, but were fasted for 8 hrs prior to the study. Animal protocols were approved by the Sun Yat-Sen University Animal Care Committee, and the experiments were performed with adherence to the guidelines provided by the National Institutes of Health for the use of animals in laboratory experiments.

### 2.2. Orthotopic Autologous Liver Transplantation (OALT) Model

Rats were sedated with intraperitoneal injection of pentobarbital (30 mg/kg) under 50% oxygen delivered using an animal mask. A standard OALT model was created as previously published [31, 32]. The entire operation procedures were aseptic. The model simulates the main hemodynamic processes during perioperative period of liver transplantation, in which the anhepatic phase can be controlled without inducing the complications of rejection or other nonsurgical factors in orthotopic liver transplantation using the cuff technique.

### 2.3. Hemin and ZnPP

Hemin and ZnPP (Sigma-Aldrich, St. Louis, MO) were solubilized in diluted solution containing sodium hydroxide (0.1 mol/L); hydrochloride acid was used to lower the pH to 7.4 and then diluted to 5 mg/mL (Hemin) and 4 mg/mL (ZnPP) using 0.85% saline solution, respectively.

### 2.4. Animals Groups

Six experimental animal groups (*n* = 3–6/group) were established with random animal selection: sham + saline, sham + hemin and sham + ZnPP, OALT + saline, OALT + hemin, and OALT + ZnPP. Rats were intraperitoneally injected with saline, hemin (30 mg/kg), and ZnPP (20 mg/kg) separately in designated groups 24 h before operation and then received celiotomy and vascular separation with or without OALT at 8 hours after OALT. We assumed no significant discrepancy between two sham groups with respective ZnPP and hemin pretreatment; the results were pooled from two groups (sham + hemin) and (sham + ZnPP) and were merged as a single sham group.

### 2.5. Collection of Intestinal Mucosa

The animals were sacrificed with an overdose of pentobarbital (200 mg/kg i.p.) at 8 h after OALT. We carefully removed the entire small intestine and cut a 0.5 cm length of intestine that was approximately 10 cm distance from the end of ileum. We fixed the tissue in 4% (concentration) formalin diluted in PBS and then paraffin-embedded it for section. We completely washed the remaining small intestine with 0.9% sodium chloride that was precooled at 4°C and then exposed the intestinal epithelium by cutting one side of the wall linearly. We rinsed the opened epithelium with precooled 0.9% sodium chloride and dried it by blotting off the remaining moisture with filter paper. We harvested the mucosal epithelium by gentle separation of it from the wall with a glass slide within a plate on the ice and then it was stored at −80°C for further analysis [33].

### 2.6. Histological Examination

Sections at five *μ*m thickness were cut from the blocks described above and stained with hematoxylin-eosin (H&E) for viewing histologic changes. Two pathologists who were blind to coded samples scored the mucosal inflammation and injury and average core for each sample was used for comparison [33].

### 2.7. Intestine Malonaldehyde (MDA) Concentration and Superoxide Dismutase (SOD) Activity

Intestinal mucosa was homogenized with normal saline. The MDA, a lipid peroxidation end product in the tissue with oxidative stress, was TBA assayed (Jiancheng Bioengineering Ltd., Nanjing, China). The MDA level in intestine was expressed as millimole/mg (protein). Once intestine tissue homogenate was ready, it was centrifuged for 15 min at 3000 g after a 5 min incubation at −20°C. The supernatant was separated from pellet and used for SOD activity detection by SOD detection kit (Jiancheng Bioengineering Ltd., Nanjing, China). The activity of SOD in intestinal mucosa was calculated as U/mg (protein) [5].

### 2.8. Enzyme-Linked Immunosorbent Assay

D-Lactic acid (D-LA, Biosamite Biotechnology Co. Ltd., China), diamine oxidase (DAO), and intestinal-type fatty acid-binding protein (FABP2) (both from Cloud-Clone Corp., USA) in serum and IL-6 and TNF-*α* levels in intestinal mucosa were measured following the standard ELISA procedure (Jiancheng Bioengineering Ltd., Nanjing, China).

### 2.9. Immunohistochemical Assay for Cleaved Caspase-3

The 5 *µ*m sections cut from the tissue blocks were subjected to standard procedure for dewaxing, blocking endogenous peroxidase and exposing antigenic sites before immunohistochemical staining. Mouse anti-cleaved caspase-3 antibody (Santa Cruz, CA) was diluted at 1 : 200 as primary antibody. Positive signal was visualized with 3,3′-diaminobenzidine (DAB, Dako Cytromation, USA) color reaction. Nuclei were stained with hematoxylin. Under the code five fields per each slide at random choice of the viewer were semiquantified.

### 2.10. Immunofluorescence

Briefly, potential nonspecific staining in the sections was blocked with 5% bovine serum albumin and 0.3% Triton X-100 in PBS. Mouse anti-Nrf2 (1 : 100) (Abcam, UK) or anti-NF-*κ*B (1 : 100) (Abcam, UK) antibodies were used as primary antibodies and then followed by a secondary antibody conjugated with fluorescence (1 : 100) (Life technologies, USA). Fluorescent microscope (Leica, DMLB2, Germany) was utilized for viewing the stained sections.

### 2.11. Western Blot Assay

We followed the standard protein separation and blotting procedures. Monoclonal antibody to zonula occludens-1 (ZO-1), occludin, HO-1, and *β*-actin (Santa Cruz) were used as primary antibodies, and the HP-conjugated anti mouse IgG (Cell Signaling Technology) as secondary antibody. Protein bands were detected by ECL kit (enhanced chemiluminescence detection KGP1125, Nanjing KeyGen Biotech. Co., Ltd.); *β*-actin band density was used as loaded sample reference to normalize relative level of each detected protein [32].

### 2.12. TUNEL Staining

Apoptosis in the intestine sections was examined after TUNEL staining with in situ cell death detection kit (Roche, Basel, Switzerland). The DAPI (Invitrogen) was used to stain nuclei. The average number of apoptotic cells was calculated from five random fields.

### 2.13. Statistical Analysis

Quantitative data are presented as mean ± standard error. Each sample was analyzed in triplicate for all biochemical assays. Therefore, all the data were means of triplicate measurements. Significance was evaluated using* one-way ANOVA* test* (SPSS 13.0*, SPSS Inc., Chicago, III) followed by* Tukey post hoc* multiple comparisons test for unpaired values. Statistical significance was called when *p* < 0.05.

## 3. Results

### 3.1. Histopathological Analyses of Intestines in Animals with OALT

**Figure 1:**
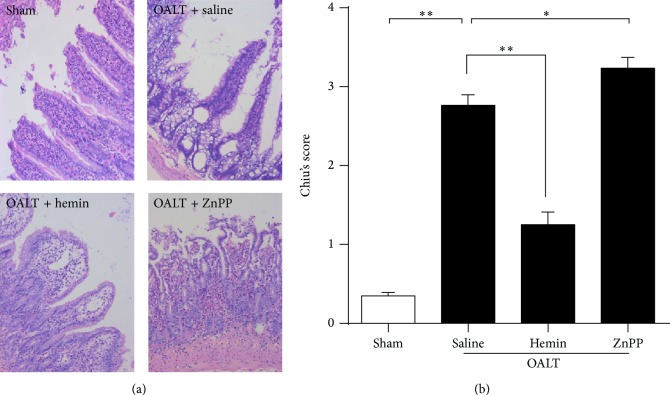
Histopathologic changes in intestines after orthotopic autologous liver transplantation (OALT). HE-stained intestine sections collected at 8 h after reperfusion from the sham, OALT + saline, OALT + hemin, and OALT + ZnPP groups (a) (×200). Rats were intraperitoneally injected with saline, hemin (30 mg/kg), and ZnPP (20 mg/kg) separately in corresponding groups 24 h before operation and then received celiotomy and vascular separation with or without OALT. Intestinal mucosa injury was graded by Chiu's score (b). The data were presented as the mean ± SD, *n* = 3–6 per group. ^∗^ *p* < 0.05, ^∗∗^ *p* < 0.01,* one-way ANOVA* with* Tukey test*.

In order to assess the histopathological changes induced with the treatment protocols, two pathologists independently scored the intestinal mucosa injury for each of coded samples and average score was used for analysis (Figure 1(b)). Results showed that a serious intestine injury occurred at 8 h after OALT (*p* < 0.01 versus sham). OALT procedure caused cytopathological changes featured with necrosis and inflammation in the intestine mucosa in group pretreated with saline. However, the intestinal injury score was sharply decreased in the animals pretreated with a HO-1 activator hemin in OALT + hemin group (*p* < 0.01 versus OALT + saline). Further, the injury score, in the animals pretreated with HO-1 inhibitor in OALT + ZnPP group, was elevated comparing the score in OALT + saline group (*p* < 0.05 versus OALT + saline).

### 3.2. Restoration of Intestinal TJ Was Accompanied by Elevated HO-1 Expression

Since epithelial TJ regulates paracellular permeability the expression of ZO-1 and occludin, selected from TJ proteins, was analyzed by Western blotting (Figure 4). OALT reduced the intestinal ZO-1 and occludin expression, accompanied by an increase in HO-1 expression. Treatment with hemin at 24 h before operation significantly elevated HO-1 protein level comparing OALT-induced HO-1 increase. We investigated whether elevation of HO-1 expression can protect the intestinal cells against OALT-induced barrier dysfunction. OALT-induced reduction of ZO-1 and occludin were mitigated by the hemin pretreatment (*p* < 0.05 versus OALT + saline). Conversely, after treatment with ZnPP, the ZO-1 and occludin levels were lower than those in OALT + saline (*p* < 0.05).

### 3.3. The Elevation of HO-1 and Restoration of Intestinal Barrier Function after OALT

**Figure 2:**
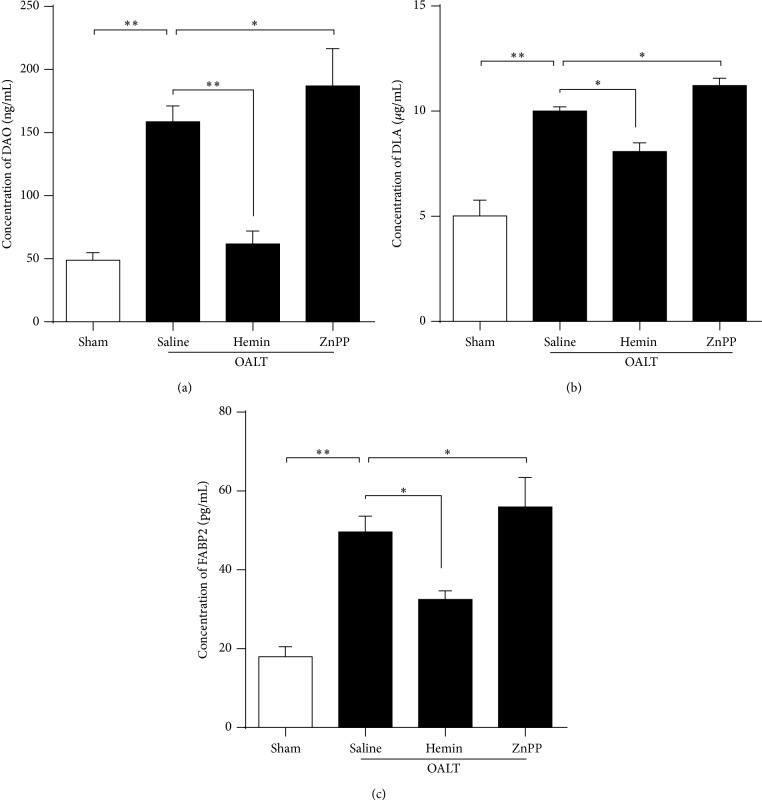
The impact of HO-1 expression level on intestinal barrier function after OALT. Concentrations of diamine oxidase (DAO) (a), D-lactic acid (D-LA) (b), and intestinal fatty acid-binding protein (FABP2) (c) in serum were detected to determine intestinal epithelial function. The serum was collected from each animal in all groups at 8 h after reperfusion. The results were presented as the mean ± SD, *n* = 3–6 per group. ^∗^ *p* < 0.05, ^∗∗^ *p* < 0.01,* one-way ANOVA* with* Tukey test*.

Concentrations of D-lactic acid (D-LA) (Figure 2(a)), diamine oxidase (DAO) (Figure 2(b)), and intestinal-type fatty acid-binding protein (FABP2) (Figure 2(c)) were used as biomarkers to assess the integrity of intestinal epithelia in this study. Significantly higher serum D-LA, DAO, and FABP2 levels were detected in OALT animals pretreated with saline than those without OALT (*p* < 0.05 versus sham). Statistically significant lower serum D-LA, DAO, and FABP2 levels were detected in the group that received 30 mg/kg of hemin 24 h before operation compared to OALT + saline animals (*p* < 0.05). As expected, the rats received 30 mg/kg of ZnPP showed higher levels of D-LA, DAO, and FABP2 than those in OALT + saline group (*p* < 0.05).

### 3.4. Elevation of HO-1 Expression Protected the Intestine Epithelial Cells from Apoptosis Caused by OALT

**Figure 3:**
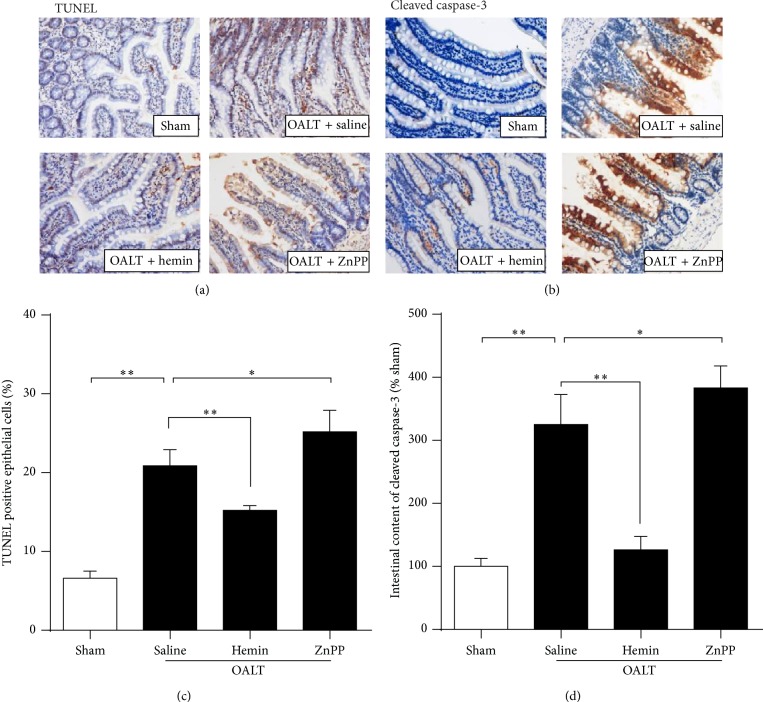
Elevation of HO-1 expression protected the intestine epithelial cells from apoptosis caused by OALT. Immunohistochemical staining of intestine sections in all 5 groups at 8 h after reperfusion for analysis of apoptosis using TUNEL assay (a); the expression of cleaved caspase-3 (b); the number of apoptotic cells from TUNEL staining (c); the expression levels of cleaved caspase-3 from immunohistochemical staining (d). The results were expressed as the mean ± SD, *n* = 3–6/group. ^∗^ *p* < 0.05, ^∗∗^ *p* < 0.01,* one-way ANOVA* with* Tukey test*.

**Figure 4:**
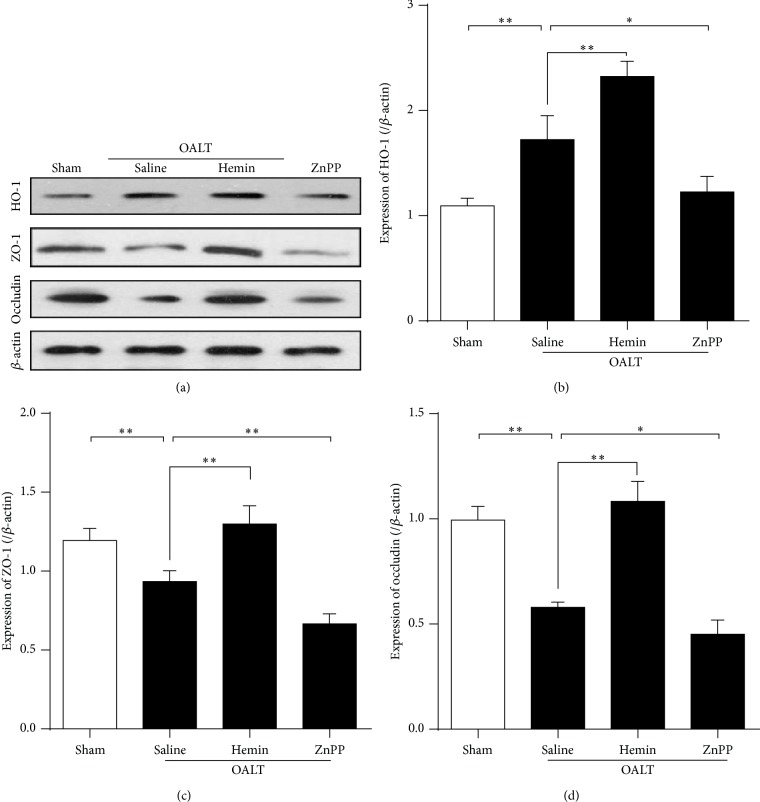
Elevated expression of HO-1 was related to the restoration of intestinal tight junction. Representative Western blots analysis (a) of the expression of HO-1 (b), ZO-1 (c), and occludin (d) in intestinal mucosa collected at 8 h after reperfusion and from each of 5 groups. *β*-actin was included as an internal standard for each blot. The results were expressed as the mean ± SD, *n* = 3–6/group. ^∗^ *p* < 0.05, ^∗∗^ *p* < 0.01,* one-way ANOVA* with* Tukey test*.

To further investigate apoptosis, the fragmented DNA and the cleaved caspase-3 expression were detected in the sections stained with TUNEL assay (Figure 3). Hemin (20 mg/kg) pretreated regressed DNA fragmentation (Figures 3(a) and 3(c)) and reduced the cleaved caspase-3 expression level in comparison to group OALT + saline (Figures 3(b) and 3(d)), implying that OALT-induced apoptosis in the epithelia could be mitigated by HO-1 activation. Furthermore, ZnPP (30 mg/kg) aggravated the apoptosis in the epithelial cells after OALT, evidenced by significantly more fragmented DNA and cleaved caspase-3 expression in intestine than that in OALT + saline group (*p* < 0.05).

### 3.5. The HO-1 Protective Function Was Related to the Activation of Transcription Factor Nrf2 and Reduction of NF-*κ*B

**Figure 5:**
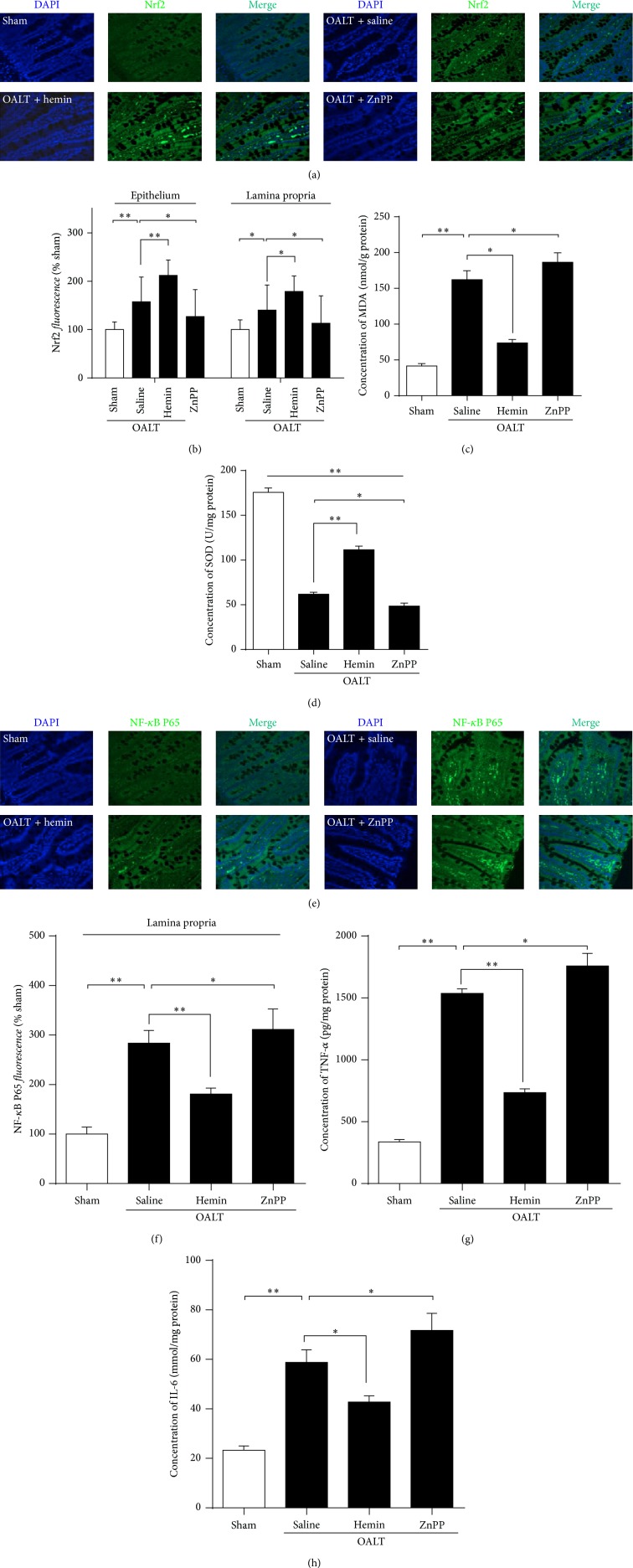
The HO-1 protective function and the translocation of transcription factors NF-*κ*B and Nrf2. Immunofluorescent staining of Nrf2 (a) and NF-*κ*B (e) using intestinal sections harvested at 8 h after reperfusion from 5 groups. And fluorescence quantitation of Nrf2 in intestine epithelium and lamina propria (b) and NF-*κ*B in lamina propria (f) was carried out. Malonaldehyde (MDA) (c), the end product of lipid peroxidation, was detected by TBARs method from the intestinal mucosa. The activity of superoxide dismutase (SOD) (d) that acts as an important antioxidant defense in intestine cells exposed to reactive oxygen species (ROS) was also detected by ELISA assay. The NF-*κ*B downstream inflammation cytokines TNF-*α* (g) and IL-6 (h) were also detected by ELISA assay. *β*-actin was included in parallel as loading reference. The results were expressed as the mean ± SD, *n* = 3–6/group. ^∗^ *p* < 0.05, ^∗∗^ *p* < 0.01,* one-way ANOVA* with* Tukey test*.

Nrf2/ARE/HO-1 pathway is closely involved in protecting liver transplantation-induced remote organ injury. Sections from the sham group contained a few nuclear Nrf2-positive cells in epithelium lamina propria after immunofluorescent staining (Figures 5(a)-5(b)); the prominently increased nuclear Nrf2-positive cells in the saline intestinal tissue (epithelium and lamina propria) were detected, accompanied by elevation of the end product of lipid peroxidation malonaldehyde (MDA) (Figure 5(c)). The hemin treatment significantly activated the Nrf2 nuclear translocation in intestine epithelium and lamina propria and increased the activity of superoxide dismutase (SOD) of mucosa (Figure 5(d)) at 8 h after OALT. However, ZnPP treatment significantly inhibited the translocation of Nrf2.

Liver transplantation-induced remote organ injury is largely NF-*κ*B pathway dependent. The activation of NF-*κ*B was also determined by immunofluorescent staining. The nuclear transportation of NF-*κ*B in intestine lamina propria and its downstream cytokines IL-6 (Figure 5(g)) and TNF-*α* (Figure 5(h)) in intestinal tissue of the OALT + hemin group were markedly lower (Figure 5(e)) than those in the OALT group. More translocations were observed in OALT + ZnPP group than hemin group, suggesting that HO-1 activation inhibits chain inflammation reactions.

## 4. Discussion

Intestine injury associated with liver transplantation can result in bacterial or endotoxin translocation in both the experimental and clinical setting [34–36]. The physiopathological changes in liver transplantation-induced intestinal injury include cytotoxicity and pathological changes in the intestinal mucosa barrier. Moreover, the intactness of gastrointestinal tract could have profound impact on functionality of other organs as GI has been called “motor of multiple organ failure.” Such interdependence makes liver the most vulnerable organ after intestinal injury because liver and intestine share the anatomical common pathway such as coupled vasculature [37]. Restoration of intestine barrier function through protecting the barrier structure integrity may facilitate recipients recovering from liver transplantation procedure that causes systemic changes in physiopathology. However, the detailed mechanisms of intestinal barrier dysfunction induced by liver transplantation remain much unknown.

We investigated intestinal barrier dysfunction in orthotopic autologous liver transplantation (OALT) and the protection from injury by hemin-elevated HO-1 expression in this study. Severe intestinal mucosal injury appeared at 8 h after OALT. The falling of epithelial cells, breaking of villi, fusing of adjacent villi, mucosal atrophy, and edema were among main pathological changes in the intestinal mucosa. Intestinal HO-1 protein expression level turned higher after OALT than that in the sham group, indicating that upregulation of HO-1 expression might represent an intrinsic protection against injury after OALT. These results were consistent with additional HO-1 upregulation induced by the pretreatment with hemin, resulting in the detectable mitigation of systemic inflammatory response and oxidative stress. HO-1 activation induced protective function was further supported by zinc-protoporphyrin (ZnPP) treatments, which inhibited HO-1 expression in the intestinal epithelia, leading to a severer intestinal mucosa injury after rats receiving OALT. All data suggest a protective function of HO-1 in the event of injury. Hemin has been widely used as a pharmacological agent to upregulate HO-1 expression to mitigate injury upon inflammation [38]. The negative impact on HO-1 protective property in presence of HO-1 inhibitor ZnPP is in agreement with the published observations that the ZnPP inhibition of HO-1 function [39] abolished the HO-1 beneficial effects.

Next, we dissected the detailed mechanism underlying the intestine injury. The intestinal mucosa is highly susceptible to ischemia and hyperpermeability can result from the injury of mucosa. The structure of TJ and epithelial barrier is maintained essentially through the interaction between occludin and ZO-1 [40]. In this study, we detected significantly lower occludin and ZO-1 expression in intestinal epithelia at 8 h after OALT. The lower expression of occludin and ZO-1 was accompanied by increasing concentrations of D-LA, DAO, and FABP2 in serum, which clearly suggested a compromised TJs structure leading to the epithelial barrier dysfunction. Furthermore, using HO-1 inhibitor ZnPP and agonist hemin pretreatment, we provided the evidence that increased expression of HO-1 leads to protection of TJs against disrupting intestinal epithelium. This observation is consistent with the finding that elevating HO-1 expression can facilitate the synthesis of TJ proteins in the epithelial/endothelial cell, preventing TJs from disruption [41].

Moreover, OALT elevated intestine oxidative stress level led to production of higher level MDA that could increase paracellular permeability [42]. TNF-*α*, an inflammatory cytokine, which was subjected to a qualitative change observed in the TJ and redistribution of ZO-1 [43], was significantly decreased by the intervention with hemin compared with saline in rats receiving OALT in our study. Therefore, we tested the underlying signal pathway for HO-1 activation. We found that HO-1 activation was accompanied by the reduction of lipid peroxidation product MDA and TNF-*α* and IL-6 cytokines, which reduced the inflammatory reaction and mitigated the damage to OALT-induced intestinal barrier.

HO-1 activation has its ability to scavenge intracellular ROS and contributes to reduction of oxidative stress. Pretreatment with HO-1 agonist hemin reduced the OALT-initiated ROS production and barrier disorder and elevated the expression of ZO-1 and occludin. The protective benefit of elevated HO-1 expression was also associated with more effective Nrf2 translocation, which functions as a main molecule defending the cytotoxicity of oxidative stress [44]. Nrf2 translocation led to expression of downstream antioxidant enzymes including HO-1. In this study a more frequent Nrf2 nuclear translocation in mucosa was detected in hemin pretreatment group than that in control OALT group, accompanied by increased SOD activity and decreased NF-*κ*B translocation, which upregulates IL-6 and TNF-*α* expression. Taken together, these findings suggest that the oxidative stress likely mediated the barrier dysfunction under OALT circumstances. Our findings are consistent with published data that antioxidants can inhibit H_2_O_2_-induced paracellular hyperpermeability [45]. Meanwhile, the reduced TJ protein expression has been proved to play an important role in intestinal permeability in our study, which was consistent with others' study on intestine disease [46, 47]. However, as tight junction localization alteration might be more direct in studying the tight junction function [48],* in vitro* study will be carried out to explore the influence of inflammation and oxidative stress on tight junction localization and its underling mechanisms by using intestinal epithelium cell culture. On the other hand, although we found that HO-1 activation mitigated OALT-induced intestine injury, probably via restoring efficient expression of TJ proteins, additional studies investigating the interaction between HO-1 and TJ proteins are warranted. As hemin was only administrated before ischemia, it is interesting to investigate impacts of hemin treatment initiated after the onset of ischemia or during reperfusion.

## 5. Conclusions

**Figure 6:**
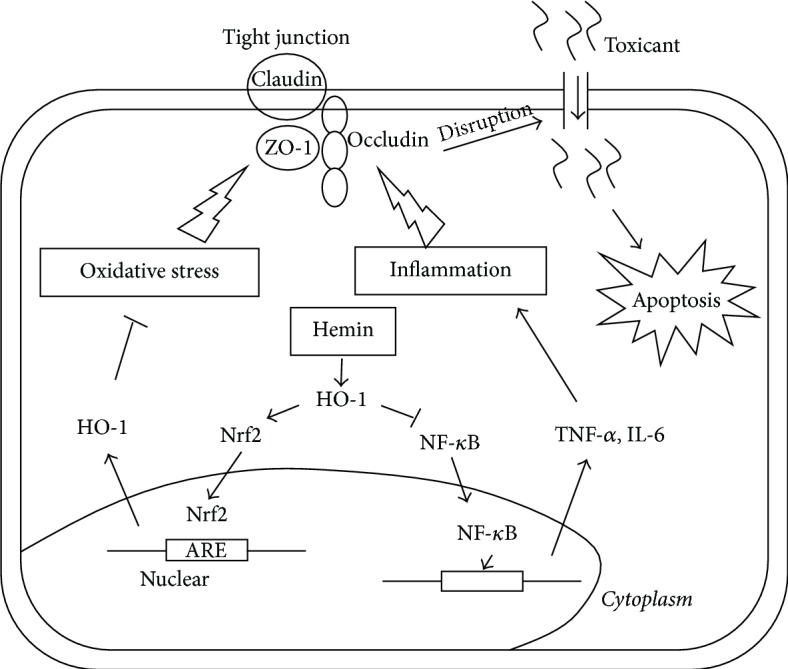
An illustration of HO-1 protection of intestinal barrier function. Intestinal inflammation and oxidative stress were induced by OALT, leading to the dysfunction of intestinal barrier that is primarily composed of tight junction (TJ). Elevation of HO-1 expression by hemin pretreatment could restore the TJ function to prevent the external toxicant from entering into cells. Cytotoxicity may cause cell apoptosis. These protective effects were associated with reduction of intercellular oxidative stress and tissue inflammation via activation of Nrf2/ARE pathway and inhibition of NF-*κ*B pathway.

Our study showed that intestinal barrier injury and dysfunction were triggered by OALT. Furthermore, our study provided the evidence that elevated expression of HO-1 in intestine can improve the pathological outcomes of OALT-related oxidative stress and ischemia on mucosal barrier structure and function (Figure 6). Our results offered new insights into protective function of HO-1 in liver transplantation-induced intestinal injury. In addition, the involvement of inflammation and oxidative stress indicates that anti-inflammatory agents and antioxidants may relieve adverse effects from liver transplantation on the small intestine.
